# Supplementary material for: TNFSF14-Derived Molecules as a Novel Treatment for Obesity and Type 2 Diabetes
Source: Int J Mol Sci. 2021 Sep 30;22(19):10647. doi: 10.3390/ijms221910647 (PMC8508965; doi:10.3390/ijms221910647)
Supplement: Supplementary file 1 [file ijms-22-10647-s001.zip › ijms-1372921-supplementary.pdf]

Supplementary materials

TNFSF14-Derived Molecules as a Novel Treatment for Obesity and Type 2 Diabetes

Mark Agostino [1,2,3], Jennifer Rooney [4], Lakshini Herat [4], Jennifer Matthews [4], Allyson Simonds [1], Susan E. Northfield [5], Denham Hopper [5,6], Markus P Schlaich [4,7,8,9] and Vance B Matthews [4,*]

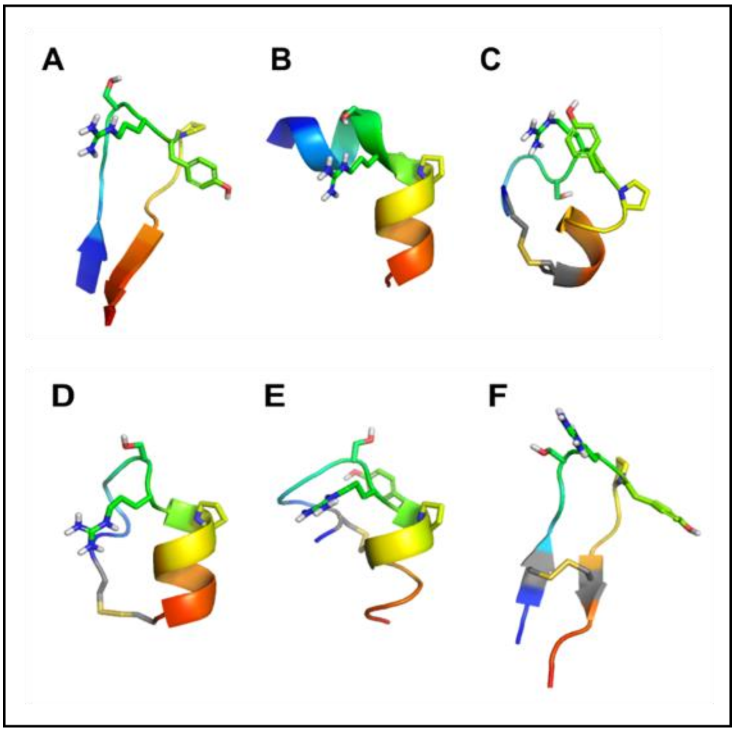

Figure S1. Optimisation of peptide **2** via introduction of disulfide crosslinks to maintain β-hairpin geometry. A. Peptide **2** conformation as in homology-modelled structure of mouse TNFSF14. B. Top-ranked conformation of peptide **2** obtained from PEP-FOLD. C. Top-ranked conformation of peptide **2.1** obtained from PEP-FOLD. D. Top-ranked conformation of peptide **2.2** obtained from PEP-FOLD. E. Top-ranked conformation of peptide **2.3** obtained from PEP-FOLD. F. Top-ranked conformation of peptide **2.4** obtained from PEP-FOLD. Conformations taken from PEP-FOLD were energy minimised in Prime. All panels coloured in red-to-blue rainbow from N- to C-terminus; disulfide crosslinks shown in grey.

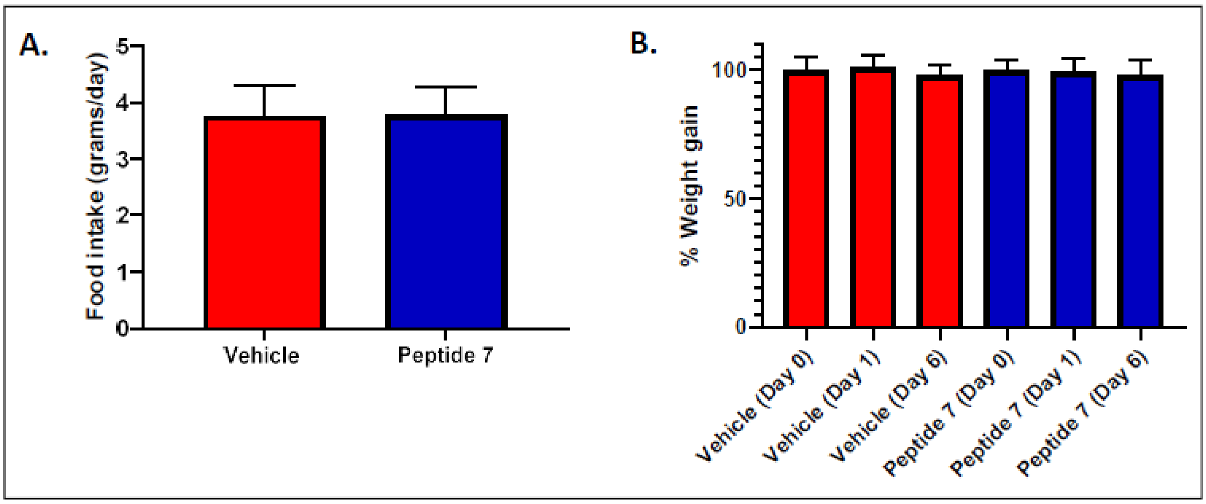

Figure S2. Peptide 7 does not affect food intake or weight loss in high fat diet fed mice. **A.** Daily food intake and **B.** Weight gain after 24hr and 6 days of treatment with vehicle or Peptide 7. Mean + SD.

Supplementary Table 1. Sequence alignments used for homology modelling.

Protein and template	Sequence alignment
mTNFSF14	EVNPAAHLTGANSSLTGSGGPLLWETQLGLAFLRGLSYHDGALVVTK
hTNFSF14 (PDB 4RSU)	QANPAAHLTGANASLIGIGGPLLWETRLGLAFLRGLTYHDGALVTME
	:.**********:**.* ********.*********:*******:
	AGYYYIYSKVQLGGVGCPLGLAS..TITHGLYKRTPRYPEELELLVS
	PGYYYVYSKVQLSGVGCPQGLANGLPITHGLYKRTSRYPKELELLVS
	.****:****** ***** ***. ********* *** *******
	QQSPCGRATSSSRVWWDSSFLGGVVHLEAGEEVVVRVLDERLV
	RRSPCGRANSS.RVWWDSSFLGGVVHLEAGEEVVVRVPGNRLV
	..******.** ************************* :***
mTNFRSF14	RSLPSCKEDEYPVGSECCPKCSPGYRVKEACGELTGTVCEPCPPGTY
hTNFRSF14 (PDB 4RSU)	SAQPSCRQEEFLVGDECCPMCNPGYHVKQVCSEHTGTVCAPCPPQTY
	. ***:::*: ** **** *.***.**:.* * ***** **** **
	IAHLNGLSKCLQCQMCDPAMGLRASRNCSRTENAVCGCSPGHFCIVQ
	TAHANGLSKCLPCGVCDPDMGLLTWQECSSWKDTVCRCIPGYFCENQ
	.**.******* * .*** *** .:** :.** * **:** *
	DGDHCAACRAYATSSPGQRVQKGGTESQDTLCT
	DGSHCSTCLQHTTCPPGQRVEKRGTHDQDTVCA
	** **:.* :.*: *****:* ** ***:*.
mLTBR	CRDQEKEYYEPQHRICCSRCPPGTYVSAKCSRIRDTVCATCAENSYN
hLTBR (PDB 4MXW)	CWDQDKEYYEPMHDVCCSRCPPGEFVFAVCSRSQDTVCKTCPHNSYN
	* **:****** * :******** :* * *** :**** **. ****
	EHWNYLTICQLCRPCDPV....EIAPCTSKRKTQCRCC.........
	EHWNHLSTCQLCRPCDIVLGFEEVAPCTSDRKAECRCQPGMSCVYLD
	****:*:.******** * *:***** **.:***
	..CT
	NECV
	*:
